# Novel Trends in Dental Color Match Using Different Shade Selection Methods: A Systematic Review and Meta-Analysis

**DOI:** 10.3390/ma15020468

**Published:** 2022-01-08

**Authors:** Louis Hardan, Rim Bourgi, Carlos Enrique Cuevas-Suárez, Monika Lukomska-Szymanska, Ana Josefina Monjarás-Ávila, Maciej Zarow, Natalia Jakubowicz, Gilbert Jorquera, Tarek Ashi, Davide Mancino, Naji Kharouf, Youssef Haikel

**Affiliations:** 1 Department of Restorative Dentistry, School of Dentistry, Saint-Joseph University, Beirut 1107 2180, Lebanon; louis.hardan@usj.edu.lb (L.H.); rim.bourgi@net.usj.edu.lb (R.B.); 2 Dental Materials Laboratory, Academic Area of Dentistry, Autonomous University of Hidalgo State, Circuito Ex Hacienda la Concepción S/N, San Agustín Tlaxiaca 42160, HGO, Mexico; ana_monjaras@uaeh.edu.mx; 3 Department of General Dentistry, Medical University of Lodz, 251 Pomorska St., 92-213 Lodz, Poland; monika.lukomska-szymanska@umed.lodz.pl; 4 “NZOZ SPS Dentist” Dental Clinic and Postgraduate Course Centre, pl. Inwalidow 7/5, 30-033 Cracow, Poland; dentist@dentist.com.pl (M.Z.); nljakubowicz@gmail.com (N.J.); 5 Department of Prosthodontics, Universidad de los Andes, Santiago 12455, Chile; gjorquera@uandes.cl; 6 Private Practice, Zahnarztpraxis Ashi, Bahnhofstr 2, 09419 Thum, Germany; tarekachi@live.com; 7 Department of Biomaterials and Bioengineering, INSERM UMR_S 1121, Biomaterials and Bioengineering, 67000 Strasbourg, France; endodontiefrancaise@outlook.com (D.M.); youssef.haikel@unistra.fr (Y.H.); 8 Department of Endodontics, Faculty of Dental Medicine, Strasbourg University, 67000 Strasbourg, France

**Keywords:** color, dental shade, digital dentistry, shade selection, smartphone

## Abstract

Since color matching is considered a subjective procedure, accurate shade choice is often the most challenging stage of recreating the natural appearance of teeth. Furthermore, accurate determination of tooth color is imperative for the final outcome of dental restorations. The purpose of this research is to assess the accuracy of color match between diverse shade selection methods throughout a systematic review and meta-analysis. Two independent investigators (L.H. and R.B.) screened the literature in five electronic databases. Randomized controlled trials or in vitro papers studying the effect of using either digital shade selection or visual shade selection on the accuracy of color match were included. A total of 13 manuscripts comprised the meta-analysis. Color difference (ΔE) between restorations where the shade matching was performed by the conventional method was greater than those where the shade matching was performed by computerized methods (*p* = 0.007). According to the subgroup analysis, only the use of digital photographs for shade matching showed a reduction in the (ΔE) (*p* < 0.0001), while the use of a spectrophotometer has no advantages over the use of visual shade guide tabs (*p* = 0.57). On the other hand, global analysis showed that incorrect shade matching was higher when the conventional method using shade guide tabs was used (*p* < 0.001), irrespective of whether a spectrophotometer or a digital camera was used (*p* < 0.001). This study concluded that the use of digital photography and spectrophotometric measurements led to fewer color differences and less incorrect shade matching than conventional methods using color shade tabs.

## 1. Introduction

The fabrication of indirect restorations necessitates worthy interaction between clinicians and laboratory technicians. Furthermore, shade choice is the often the most challenging stage in the recreation of the natural appearance of teeth [1]. According to previous studies, differences among practitioners concerning shade matching for the same teeth occur over days [2,3]. In fact, researchers have considered color matching as a subjective procedure, reliant on several influences, such as light source, object, and observer [4]. While there is no process considered as the gold standard, different methods to assess color in dentistry exist, including visual and instrumental [5].

Color communication using shade guides is the most shared technique. However, this method is considered subjective, since it is induced by age, sex, experience of the observer, eye fatigue, and ambient light [6]. In this sense, instrumental methods have gained popularity; however, they are expensive and not always available to the dentist [2,7]. It is important to note that instrumental methods include spectrophotometers, scanners, cross-polarizing filters, digital cameras, and smartphones [2,8]. These devices consist of a detector, signal conditioner, and software that process the signal to make the data usable in a clinic or laboratory [9]. Spectrophotometric color measurements are capable of reliably quantifying the color of both extracted teeth and dental materials [10].

Digital photographs are a common interaction tool in dental workplaces [11]. Digital photographs are able to capture clear images of the tooth and have been progressively used to document the whole clinical procedure [2,12]. Predictably, digital photographs of natural teeth can show noteworthy color modifications when the optimal conditions of light illumination are not used [12].

On the other hand, there has been an increased awareness for aesthetically pleasing restorations among patients. Hence, it is a practitioner’s responsibility to provide restorations which can adequately mimic the surrounding natural dentition [13]. As scientific evidence comparing differences in visual shade matching using different devices is scarce, this topic remains controversial among researchers. Therefore, the purpose of the present study is to systematically review the literature to compare the accuracy of color matching between different shade selection methods. The null hypothesis tested was that all different shade selection methods would have the same accuracy.

## 2. Materials and Methods

This systematic review and meta-analysis were implemented in agreement with the PRISMA instructions [14]. The registration protocol was carried out in Prospero with the registration number CRD42021288077. The resulting PICOS framework performed is described in Table 1. The research question was: “Is the use of spectroscopic or camera method in dentistry accurate for color selection of the tooth?”

### 2.1. Literature Search

A literature search was conducted by two independent authors (L.H. and R.B.) up to July 15, 2021. Different electronic databases were selected: PubMed MedLine, Scopus, ISI Web of Science, Cochrane library, and EMBASE. These databases were used for identifying articles that could fit the specific criteria. The keywords, as well as the search strategy performed in PubMed, are summarized in Table 2. The search strategies for Scopus, ISI Web of Science, Cochrane library, and EMBASE databases are presented as Supplementary Materials (Tables S1–S4). The investigators manually checked the list of references of each included paper for the discovery of additional manuscripts. After the search, the articles were introduced into Mendeley Desktop 1.17.11 software (Glyph & Cog, LLC, London, UK) to eliminate duplicates.

### 2.2. Study Selection

Two researchers (L.H. and R.B.) independently assessed the abstracts and titles of all the articles. Studies for full-text review were chosen based on the following eligibility criteria: (1) randomized controlled trial or in vitro papers studying the effect of using either digital shade selection or visual shade selection on the accuracy of color selection; (2) publishing in the English language; (3) assessing the accuracy of color measurement of the tooth and/or restorative material; (4) including a numerical value for the difference in color of the subject selected. Case series, reviews, case reports, and pilot studies were excluded. Full versions of any potentially suitable manuscripts were analyzed. Manuscripts that met the inclusion conditions or had inadequate data in the abstract and title to provide a clear judgment were considered for full evaluation. Full-text papers were evaluated by independent reviewers. Any variations in the decision-making process in regard to the suitability of the accepted articles was resolved and decided upon through the consensus of a third author (C.E.C.-S.). Only manuscripts that satisfied all of the eligibility criteria recorded were integrated for assessment.

### 2.3. Data Extraction

The information of concern from the papers included was organized using a harmonized form in a Microsoft Office Excel 2019 spreadsheet (Microsoft Corporation, Redmond, WA, USA). These records comprised demographic data (author and year of publication), method used for shade match, materials used, type of room light, the main outcome, and main results. The corresponding authors of the included articles were communicated twice via e-mail to regain any missing data. If the investigators did not answer within 10 days of the first communication, the omitted data was not incorporated.

### 2.4. Quality Assessment

The methodological quality of each involved manuscript was independently assessed by two investigators (R.B. and L.H.), considering the parameters of the Cochrane Collaboration’s tool for evaluating risk of bias in randomized trials [15]. For the in vitro articles, the risk of bias in each manuscript was assessed according to the description of the subsequent parameters: specimen randomization, single operator, operator blinded, control group, standardized specimens, and sample size calculation [15]. If the reviewers stated the parameter, the study expected a “YES” of that precise parameter. In case of missing information, the parameter received a “NO.” The risk of bias was categorized conferring to the sum of “YES” answers received: 1 to 2 designated a high bias, 3 to 4 medium, and 5 to 6 showed a low risk of bias. For the clinical studies, the risk of bias in each paper was considered according to the description of the subsequent parameters: allocation concealment, incomplete outcome data, sequence generation, blinding of the outcome assessors, selective outcome reporting, and other potential sources of bias. All these areas were assessed at the analysis level. Through risk of bias evaluation, any discrepancies between the researchers were solved through argument by accessing a third referee (C.E.C.-S.).

### 2.5. Statistical Analysis

Meta-analyses were completed using the Review Manager Software version 5.3.5 (The Nordic Cochrane Centre, The Cochrane Collaboration, Copenhagen, Denmark). For the quantitative data, a random-effect model was conducted for evaluates, and pooled-effect estimates were acquired by comparing the standardized mean difference of the (ΔE) between the classical shade guide and the computerized methods. Subgroup analyses were accomplished using a spectrophotometer or a digital camera to assess the shade selection. For the qualitative data, global analysis was conducted using a fixed-effects model, and pooled-effect estimates were acquired by comparing the risk difference of incorrect shade matching of the computerized methods (use of spectrophotometer or digital camera), or the conventional visual shade guide. A *p*-value of <0.05 was considered statistically significant. Statistical heterogeneity of the treatment effect among studies was evaluated using the Cochran Q test and the inconsistency I^2^ test.

## 3. Results

**Figure 1:**
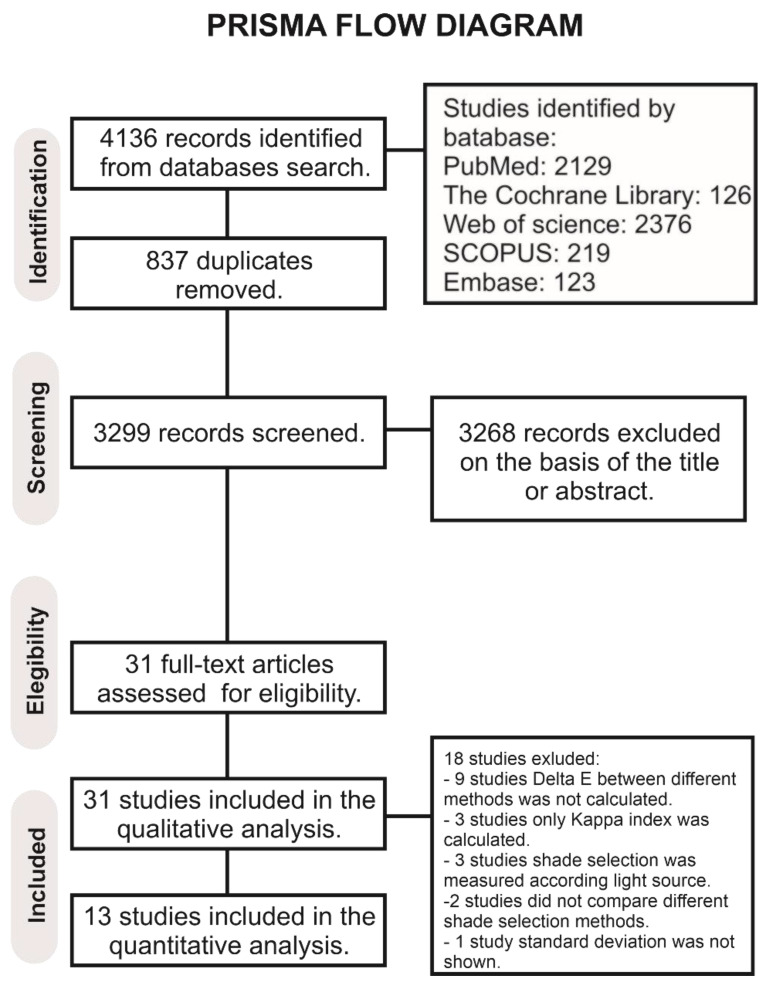
Prisma flow diagram of the research.

A total of 4136 papers were obtained from the databases (Figure 1). After removing the duplicates, the total quantity of literature found was 3299 publications for the primary examination. Then, 3268 manuscripts were excluded after revising the titles and summaries, leaving a total of 31 articles to be assessed for full-text review. Of these, 18 studies were excluded [16,17,18,19,20,21,22,23,24,25,26,27,28,29,30,31,32,33]. Exclusion reasons are assumed in the PRISMA flow diagram of the research, which resulted in a total of 13 manuscripts in the meta-analysis [34,35,36,37,38,39,40,41,42,43,44,45,46]. These manuscripts included seven in vitro studies and six clinical trials.

The characteristics of the manuscripts incorporated in this systematic review were recapitulated in Table 3.

**Figure 2:**
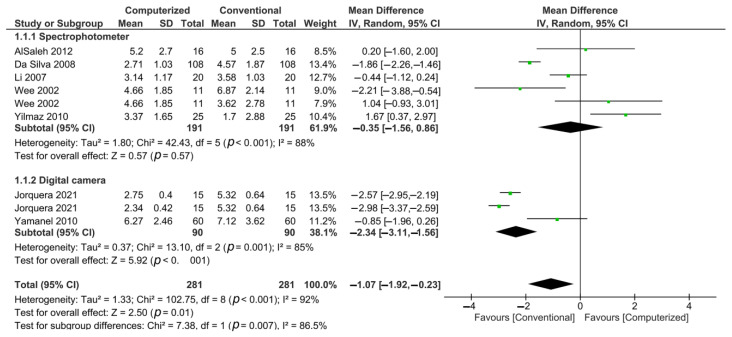
Forest plot of the quantitative analysis.

The meta-analysis of the quantitative data indicates that (ΔE) between restorations where the shade matching was performed with the conventional method was greater than those where the shade matching was performed with the computerized methods (*p* = 0.007). According to the subgroup analysis, only the use of digital photographs for shade matching showed a reduction in (ΔE) (*p* < 0.001), while the use of a spectrophotometer had no advantages over the use of visual shade guide tabs (*p* = 0.57) (Figure 2).

**Figure 3:**
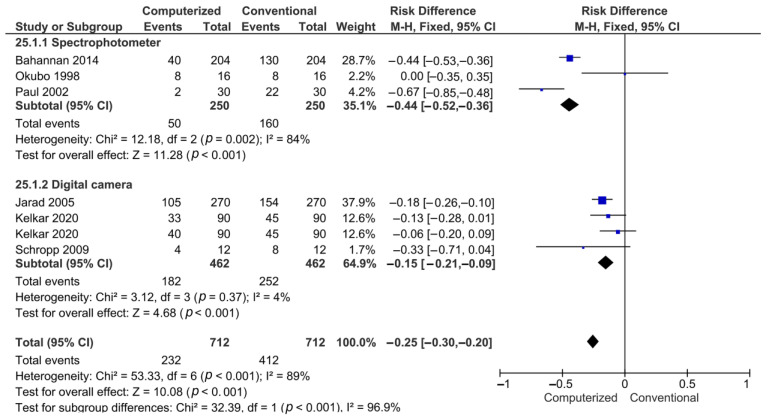
Forest plot of the qualitative analysis.

On the other hand, for the qualitative analysis, global analysis proved that incorrect shade matching was higher when the conventional method using shade guide tabs was used (*p* < 0.001), irrespective of whether a spectrophotometer or a digital camera was used (*p* < 0.001) (Figure 3).

**Figure 4:**
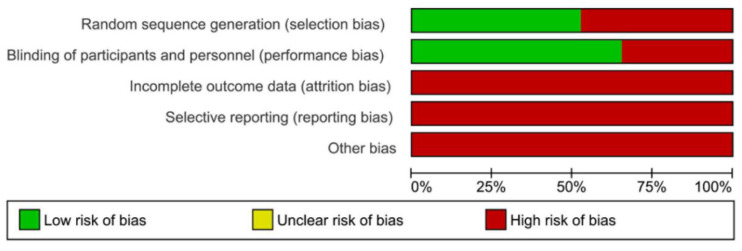
Qualitative synthesis for clinical trials.

Studying the methodological quality assessment parameters, most of the manuscripts involved were counted as having a medium risk of bias (Table 4 and Figure 4). However, several studies analyzed failed to account for the sample size calculation, single operator, and operator blinded parameters. Regarding the clinical studies, most of them were categorized as having a high risk of bias, since most of them failed to avoid performance and detection bias, reporting bias, bias due to incomplete data, and other bias.

## 4. Discussion

This systematic review and meta-analysis were directed to compare different computerized methods with the visual shade guides for shade matching. The overall findings revealed that the color differences of restorations performed using a visual shade guide were higher than with digital photography. In addition, the proportion of incorrect shade matching was higher when the visual shade guide tabs were used. Considering this, the null hypothesis stating that both digital and visual shade matching of the tooth were comparable was rejected.

It should be noted that shade matching in esthetic restorations was a key factor in the attainment of a perfect tooth color [47,48]. Furthermore, color mismatch in ceramic restorations can be very stressful for both clinicians and patients [49]. In this sense, the combination of visual and digital techniques has been suggested for a proper tooth color matching [35]. The visual technique consists of the use of shade guides to acquire the closest match to a natural tooth [5,7]. This way is subjective, and its precision is affected by several factors including experience, sex, and the need for training of the spectator, in addition to the tooth shade and light source [50,51,52]. Dental technicians should rely on a color match approach when operating only from an instruction based on a shade guide [53]. For this reason, the digital technique has been deemed more reliable than the visual technique alone [7]. Digital images achieved imperative data about tooth color crossways and the shape, surface, and characteristic features that the dental technician needs [35]. This could be explained by the improvement in communication and color selection [54]. Whenever feasible, both techniques should be combined [5,54].

In addition, mobile dental photography (MDP) seems to be favorable in terms of software applications, cost effectiveness, enhanced functionalities, and high-resolution photographs, which makes it suitable for acquiring color references [54,55]. The use of a smartphone with the help of the cross-polarizing filter of the smile Lite MDP allowed users to compare and evaluate the color matching amongst shade tabs [54,56,57].

One should bear in mind that by using this filter, the intrinsic shade variations of natural teeth for shade analysis might be revealed. In addition, it can help remove unwanted reflections and diffuse light created by the flash which can obscure features in the dental teeth, and hence produce issues in communication with the dental technician [18,21]. Consequently, referring to the results of Sampaio et al., [27] the use of cross-polarizing filters was the most standardized method for both communication and color assessment. It is important to mention that non-polarized tooth color images offered numerous glare designs on the surface of the tooth, which could be affected by the uneven distribution of saliva on tooth surfaces or roughness. These produce artifacts in tooth color image analysis. In juxtapose, a lack of glaring patterns was observed in the cross-polarized tooth color images [32].

It should be emphasized that (ΔE) obtained by two measurements of the same object can be interpreted as acceptable or perceptible to the human eyes [58].

Acceptability means the agreement of the color of the restoration, while perceptibility indicates the detection of the (ΔE) between both the tooth and the adjacent restoration [59]. A previous study suggested that a clinically acceptable level of visual perception was possible when (ΔE = 3.7) in the CIELab system [58]. This system contains three coordinates: L* which refers to the luminosity of the object and fluctuates from black to white; a* which indicates the chroma in the green-red axis; and the b* which represents the chroma in the axis of blue-yellow [60]. Each color has a numerical value, delivering an objective description [27]. This might clarify the outcomes of this analysis obtained by a visual technique, as the preceding paper displayed that a non-acceptable restoration match was obtained with a higher (ΔE) [35]. In this respect, clinicians should consider the digital technique when performing the shade-matching process. Digital images gave significant evidence about tooth color crossways, the surface, shape, and details that the dental laboratory technician needs [61,62]. Indeed, this could be possible when using a standardized light correcting device, thus helping to achieve a better result [59]. Both smartphones and digital cameras can promote a correct white balance by using a grey reference card with specific color coordinates to supply the color of the images in an accurate way [19,27,35]. This was considered successful in compensating the changes in tooth color triggered by various diffuser materials [19].

Additionally, a cross-polarizing filter might be assessed to eliminate specular reflections which can modify an image and lead to an imperfect analysis [27]. Hence, the use of less expensive instruments such as smartphones may enhance communication and help replicate the color in an easy, fast, and accurate way.

The percentage of dentists trusting the use of their smartphone cameras instead of professional DSLR cameras is rising exponentially because of the former’s easy access and manipulation [63].

The findings of this analysis should be reflected with caution, since not all smartphones and digital cameras available on the market were tested. In addition, the evidence should be directed towards different light source devices which can facilitate communication between the dentist, patient, and the lab technician. Future research needs to consist of guided, principally randomized controlled clinical trials, with the aim of affording better vision into the performance of several computerized approaches to color-shade-matching success. Moreover, different spectrophotometers are already available in the market, and further studies testing these devices can provide a better understanding of color matching between the tooth and the restoration in an affordable way.

## 5. Conclusions

The use of digital photography and spectrophotometric measurements led to fewer color differences and less incorrect shade matching than conventional methods using color shade tabs.

## Figures and Tables

**Table 1:** PICOS framework used.

Population	Color of the Tooth
Intervention	Spectroscopic or camera method for color determination
Control	Clinical perception using a shade tab guide
Outcome	Shade match
Study design	Randomized clinical trials and in vitro studies

**Table 2:** Search strategy used in PubMed.

#1	Color OR Color measurement OR Colorimeters OR Spectrophotometers OR spectrophotometer OR CIE L*a*b* OR Tooth Color OR Color Shade OR dental color OR spectrophotometry OR colorimetry OR color perception OR color matching OR color accuracy OR spectroradiometry OR color*
#2	Smartphone OR Mobile dental photography OR Digital camera OR dental photography OR digital photography OR DSLR camera OR Mobile camera OR Cell Phone Use OR photography OR photography* OR digital dentistry OR polarizing filter OR light OR light*
#3	Shade communication OR shade matching OR shade selection OR shade guide OR shade OR dental shade OR shade determination OR dental shade OR shade selection program OR visual shade matching
#4	#1 and #2 and #3

**Table 3:** Demographic and study design data of the incorporated manuscripts.

Study	Method Used for Shade Match	Materials Used	Type of Room Light	Main Outcome	Main Results
Jorquera, 2021	Shade guide Table Digital camera and cross-polarizing filter. Smartphone with a light-correcting device.	Human tooth	The room had ambient light between 5500 K and 6500 K. The clothing of the volunteers was covered with a neutral color cloth.	ΔE	Digital shade choice using both a digital camera and a smartphone displayed a threshold within the adequate values (ΔE < 3.7). Visual shade choice displayed an average ∆E above the level for acceptable values (ΔE > 3.7).
Mahn, 2021	Shade guide tab. Digital camera with a cross-polarized filter. Spectrophotometer	Human tooth	The room had ambient light between 5500 K and 6500 K. The clothing of the volunteers was covered with a neutral color cloth.	ΔE	No statistically significant differences within digital images and spectrophotometer. The visual shade process led to enormous variances compared to the other approaches beneath study.
Sampaio, 2019	Digital camera with ring flash or a dual-point rigid flash bracket. Cross-polarizing filter attached to a close-up flash. iPhone 7.	Human tooth	For comparison, the measurements were made in the same spots.	ΔE	Using a cross-polarizing filter consequences in more color-standardized photographs; however, using an iPhone 7 and a ring flash system resulted in fewer standardized pictures.
Zekonis, 2002	Colorimeter Shade guide Color slide photography	Human tooth	Non-specified	ΔE	At-home (10% carbamide peroxide) treatment sides were meaningfully distinctive from the in-office (35% hydrogen peroxide) treatment sides through all active treatment stages and during follow-up visits conferring to all three color assessment approaches.
Matis, 2002	Shade guide. Digital photographs Color-measuring device	Human tooth	Non-specified	ΔE	Shade guide and slide photography data displayed no meaningful variances between teeth lightened with agent with or without reservoirs.
Jarad, 2003	Shade guides. Digital camera.	Porcelain shade tabs	All background lighting in the room was maintained at a consistent level for all sessions. (Colour temperature was set at 6500 K).	CIELab	The viewers’ shade-harmonizing performance was significantly improved using the computer method compared to the conventional one.
Matis, 2000	Digital camera. Shade guide.	Human tooth	Non-specified	CIELab and ΔE	All three methods of evaluation revealed a significant difference in the tooth lightness.
Matis, 2000	Digital camera. Shade guide.	Human tooth	Non-specified	CIELab	This study suggests that when a higher concentra-tion of carbamide peroxide was used, the further the lightness value and ΔE altered.
Gómez-Polo, 2014	3DMaster Toothguide (Vita-Zahnfabrik) Easy- Shade Compact (Vita-Zahnfabrik) spectrophotometer	Human tooth	Recordings were made under fluorescent tubes with daylight and an intensity of 1200–1500 lux, in the same room under standardized lighting conditions.	Lightness Chroma Hue	This research showed differences between the measurement of color using the spectrophotometry tool and the visual shade selection technique.
Kröger, 2015	Spectrophotometer Visual color matching using a shade guide	Human tooth	A room with dimmed fluorescent ceiling light.	CIELab	The spectrophotometer provided higher reproducibility.
Wang, 2014	Spectrophotometer Optimized and Vitapan Classical shade guide	Human tooth	Northern daylight	CIELab and ΔE	An optimized shade guide improved the performance of color selection
He, 2019	Non-polarized photography Cross-polarization photography Spectrophotometer	Human tooth	Non-specified	CIELab and ΔE	Combining non-polarized photography, cross-polarization photography, and spectrophotometer approaches were considered reasonable for shade matching.
AlSaleh, 2012	VITA classical shade guide (VITA Zahnfabrik gmbh, Bad Säckingen, Germany) Visual	Human tooth	A light grey wall in a room away from all windows.	CIELab and ΔE	Analysis using the spectrophotometric shade was considered more accurate in comparison to human shade evaluation.
Baharin, 2013	Intraoral spectrophotometer machine Visual	Human tooth	Non-specified	Accuracy	This sudy revealed that for the anterior tooth, the patient’s position, lighting condition and number of readings acquired does impact the outcome of shade selection.
Bahannan, 2014	Visual method using a Vita-3D Master system Spectrophotometer	Human tooth	Daylight	Daylight illuminator (GTI Graphic Technology, NY, USA)	The conventional visual method was significantly inferior compared to the shade assessment method device.
Chitrarsu, 2017	Vita Toothguide 3D-Master Intraoral digital spectrophotometer (Vita Easyshade Advance 4.0)	Natural dentitions	Daylight, incandescent light, LED, and filtered LED.	CIELab	Vita Toothguide 3D-Master showed statistically important variances in shade matching in comparison to the intraoral digital spectrophotometer.
Da Silva, 2008	Shade guide systems Instrument-based color matching using a new spectrophotometric system	Tooth color for anterior metal ceramic restorations	Under daylight and color temperature of 6500 °K.	ΔE	For anterior metal ceramic restorations, using a spectrophotometric method is an effective device for imitating and communicating the color of the tooth.
Hein, 2016	A digital single-lens reflex camera.	Extracted human teeth	Non-specified	CIELab and ΔE	The use of a white balance reference card with acknowledged color coordinates can be suggested when diffusers are used for dental photography.
Miyajiwala, 2017	Visual method Spectrophotometer Digital photography	Human tooth	Daylight	CIELab	Clinically, for shade selection, the use of the digital photography method can appear as a viable alternative to the use of spectrophotometric method.
Li, 2007	Shade guide Shofu ShadeEye NCC colorimeter	Human tooth	Northern daylight	CIELab	The consistency of shade matching cannot be guaranteed by either the visual method or the colorimeter approach.
Pimentel, 2014	Visual (classic shade guide) Instrumental methods (spectrophotometer)	Natural tooth	Controlled illumination	Accuracy	Shade match using the instrumental method presented more agreement than shade match using the visual method.
Okubo, 1998	Vita Lumin shade guide teeth A computerized colorimeter	Ceramic shade guide teeth	Northern daylight	CIELab and ΔE	Color matching using the visual process is unpredictable. However, instrumental measurement of tooth color would deliver objective measured data to match the color of the natural teeth.
Olms, 2013	VITA Easyshade spectrophotometer	Ceramic veneer	Ceiling lighting (Philips Master TLD 36 W) and a dental lamp (KaVo Dental GmbH, Germany).	CIELab	This study confirmed that worthy outcomes in terms of the repeatability and precision were obtained with the help of the VITA Easyshade measurements.
Paul, 2002	Spectrophotometric assessment of tooth color visual determination	Human tooth	Light source (6500 K).	ΔE	Human shade assessment is less accurate and less reproducible when compared to spectrophotometric shade evaluation.
Schropp, 2008	Digital photographs with graphic computer software Conventional visual matching	Phantom head	Daylight lamps with a color temperature of 4800 K.	CIE LCh	Digital photographs and computer software were significantly more trustworthy than conventional visual approach for shade-matching analysis.
Tung, 2010	Digital camera in both automatic white balance (AWB) and custom white balance (CWB) under either light-emitting diode (LED) or electronic ring flash	Ceramic disks	The background lighting in the room was subdued and maintained at a constant level during the entire experiment.	CIELab	Digital images were more influenced by the illuminants and camera’s white balance setups when testing the reliability of color match.
Wee, 2002	Vita Lumin/Vita VMK 68 Vitapan 3D-Master/Vita Omega 900 Shofu ShadeEye-EX/Vintage Halo	Dental porcelain	Color-corrected D65 lighting.	CIELab	The largest mean ∆E was noted for the Vitapan 3D-Master system, which was considerably distinct from the Vita Lumin and Shofu ShadeEye systems.
Yamanel, 2010	Digital imaging Colorimeter	Composite resin shade guides	Two 6500-K fluorescent tubes were combined with two 2700 K fluorescent tubes.	CIELab	The mean ∆E values verified a statistically significant difference with the colorimeter method. However, there was no significant change when using the digital imaging approach.
Yilmaz, 2010	Intraoral colorimeter (shadeeye NCC) Visual shade determination	Metal ceramic	Non-specified	CIELab	Color imitation using instrumental shade method for the specimens made of metal ceramic was less accurate when compared to the visual shade approach.
Lakhanpal, 2016	Spectrophotometer Digital camera Digital camera with a polarizer	Extracted non-carious premolars	Dark room setup.	CIELab	A statistically important association was found to exist with the spectrophotometer and the polarization dental imaging modality for all CIE Lab color coordinates
Kelkar, 2020	Canon 5D camera with ISO 200 Two VITAPAN classical shade guides	VITAPAN	Daylight	Individual Matching Ability of each Observer	For obtaining an aesthetic outcome, the use of digital photographic approach was considered most accurate among the three shade selection approaches.

**Table 4:** Qualitative synthesis for in vitro articles.

Study	Specimen Randomization	Single Operator	Operator Blinded	Control Group	Standardized Specimens	Sample Size Calculation	Risk of Bias
Jarad, 2003	YES	YES	NO	YES	YES	NO	Medium
Kröger, 2015	YES	NO	NO	YES	YES	YES	Medium
He, 2019	NO	NO	NO	YES	YES	NO	High
Hein, 2016	YES	NO	NO	YES	YES	NO	Medium
Schropp, 2008	NO	NO	YES	YES	YES	YES	Medium
Tung, 2010	YES	NO	YES	YES	YES	NO	Medium
Wee, 2002	YES	NO	NO	YES	YES	NO	Medium
Yamanel, 2010	NO	NO	NO	YES	YES	NO	High
Yilmaz, 2010	YES	NO	YES	YES	YES	YES	Low
Lakhanpal, 2016	NO	NO	NO	YES	YES	NO	High
Kelkar, 2020	YES	NO	NO	YES	YES	YES	Medium

## Data Availability

The data that help the outcomes of this analysis are accessible from the corresponding authors upon reasonable request.

## Supplementary Materials

The following supporting information can be downloaded at: https://www.mdpi.com/article/10.3390/ma15020468/s1, Table S1: Search strategy used in SCOPUS; Table S2: Search strategy used in ISI Web of Science; Table S3: Search strategy used in the Cochrane Library; Table S4: Search strategy used in EMBASE.
