# Supplementary material for: Novel Trends in Dental Color Match Using Different Shade Selection Methods: A Systematic Review and Meta-Analysis
Source: Materials (Basel). 2022 Jan 8;15(2):468. doi: 10.3390/ma15020468 (PMC8778907; doi:10.3390/ma15020468)
Supplement: Supplementary file 1 [file materials-15-00468-s001.zip › materials-1491175-SI.pdf]

Table S1. Search strategy used in SCOPUS.

**Search strategy**	
# 1	TITLE-ABS-KEY(“Color” OR “Color measurement” OR “Colorimeters” OR “Spectrophotometers” OR “spectrophotometer” OR “CIE L*a*b*” OR “Tooth Color” OR “Color Shade” OR “dental color” OR “spectrophotometry” OR “colorimetry” OR “color perception” OR “color matching” OR “color accuracy” OR “spectroradiometry” OR “color*”) AND TITLE-ABS-KEY(“Smartphone” OR “Mobile dental photography” OR “Digital camera” OR “dental photography” OR “digital photography” OR “DSLR camera” OR “Mobile camera” OR “Cell Phone Use” OR “photography” OR “photography*” OR “digital dentistry” OR “polarizing filter” OR “light OR light*”) AND TITLE-ABS-KEY(“Shade communication” OR “shade matching” OR “shade selection” OR “shade guide” OR “shade” OR “dental shade” OR “shade determination” OR “dental shade” OR “shade selection program” OR “visual shade matching”)

Table S2. Search strategy used in ISI Web of Science.

**Search strategy**	
# 1	ALL=(Color OR Color measurement OR Colorimeters OR Spectrophotometers OR spectrophotometer OR CIE L*a*b* OR Tooth Color OR Color Shade OR dental color OR spectrophotometry OR colorimetry OR color perception OR color matching OR color accuracy OR spectroradiometry OR color*)
# 2	ALL=(Smartphone OR Mobile dental photography OR Digital camera OR dental photography OR digital photography OR DSLR camera OR Mobile camera OR Cell Phone Use OR photography OR photography* OR digital dentistry OR polarizing filter OR light)
# 3	ALL=(Shade communication OR shade matching OR shade selection OR shade guide OR shade OR dental shade OR shade determination OR dental shade OR shade selection program OR visual shade matching)OR "light OR light*") AND TITLE-ABS-KEY("Shade communication" OR "shade matching" OR "shade selection" OR "shade guide" OR "shade" OR "dental shade" OR "shade determination" OR "dental shade" OR "shade selection program" OR "visual shade matching")
# 4	# 1 AND # 2 AND # 3 AND # 4

Table S3. Search strategy used in The Cochrane Library.

**Search strategy**	
# 1	Color OR Color measurement OR Colorimeters OR Spectrophotometers OR spectrophotometer OR Tooth Color OR Color Shade OR dental color OR spectrophotometry OR colorimetry OR color perception OR color matching OR color accuracy OR spectroradiometry
# 2	Smartphone OR Mobile dental photography OR Digital camera OR dental photography OR digital photography OR DSLR camera OR Mobile camera OR Cell Phone Use OR photography OR photography* OR digital dentistry OR polarizing filter OR light
# 3	Smartphone OR Mobile dental photography OR Digital camera OR dental photography OR digital photography OR DSLR camera OR Mobile camera OR Cell Phone Use OR photography OR photography* OR digital dentistry OR polarizing filter OR light
# 4	# 1 AND # 2 AND # 3 AND # 4

Table S4. Search strategy used in Embase.

Search strategy	
# 1	‘Color’ OR ‘Color measurement’ OR ‘Colorimeters’ OR ‘Spectrophotometers’ OR ‘spectrophotometer’ OR ‘CIE L*a*b*’ OR ‘Tooth Color’ OR ‘Color Shade’ OR ‘dental color’ OR ‘spectrophotometry’ OR ‘colorimetry’ OR ‘color perception’ OR ‘color matching’ OR ‘color accuracy’ OR ‘spectroradiometry’ OR ‘color*’
# 2	‘Smartphone’ OR ‘Mobile dental photography’ OR ‘Digital camera’ OR ‘dental photography’ OR ‘digital photography’ OR ‘DSLR camera’ OR ‘Mobile camera’ OR ‘Cell Phone Use’ OR ‘photography’ OR ‘photography*’ OR ‘digital dentistry’ OR ‘polarizing filter’ OR ‘light OR light’
# 3	‘Shade communication’ OR ‘shade matching’ OR ‘shade selection’ OR ‘shade guide’ OR ‘shade’ OR ‘dental shade’ OR ‘shade determination’ OR ‘dental shade’ OR ‘shade selection program’ OR ‘visual shade matching’
# 4	# 1 AND # 2 AND # 3 AND # 4
